# Supplementary material for: Detection and prevalence of SARS-CoV-2 co-infections during the Omicron variant circulation in France
Source: Nat Commun. 2022 Oct 23;13:6316. doi: 10.1038/s41467-022-33910-9 (PMC9588762; doi:10.1038/s41467-022-33910-9)
Supplement: Supplementary file 1 — Supplementary Information [file 41467_2022_33910_MOESM1_ESM.pdf]

Supplementary Methods

Description of the co-infection detection algorithm

The co-infection detection consists in several steps based on a sample vcf file, a sample position-based depth bed file, a target region bed file and a lineage profile database generated with our database building pipeline (https://github.com/genepii/seqmet-db). Sample files are generated by bedtools and freebayes with our analysis pipeline, the target region bed file is provided with the pipeline and corresponds to the whole coding region in our case (https://github.com/genepii/seqmet). For a given sample, our pipeline first compares, thanks to a script, the sample vcf to each lineage vcf of the database to find a main lineage in the sample. Note that this main lineage is the one obtaining the best ratio of found out of expected variants, which is not necessarily the one with the highest allele frequency. All defining variants of the tested lineage for which the sample had a sequencing position depth higher than 100 reads are expected to be found if the tested lineage corresponds to one of the lineages contained in the sample. Then, each of these expected variants are searched in the sample vcf file considering both minor and major variants. A ratio of the number of found variants on the expected list is calculated and used to select, among the lineages for which the found variant count is higher than a cutoff, the best main lineage candidate. In the case where multiple lineages have the same ratio of supporting mutations, the one with the higher found mutation count is chosen, and if there is still a tie, the lineage is chosen in reverse alphabetical order (longest sub-lineage in most cases). A second round of this script is performed with two key differences. The defining variants of the newly found main lineage are excluded from the search, and only minor variants of the sample vcf file are compared to the tested lineage profiles. Performing this second round while excluding the main lineage-defining variants allows the search to be based only on variants specific to the putative secondary lineage when compared with the main

lineage. Finally, the main and secondary lineage, the found and expected variants, and the calculated ratio are reported in a summary file.

Determination of the optimal cutoff value for detecting the secondary lineage

The appropriate cutoff was determined empirically while training the pipeline on real data and positive controls included in each run. Based on the artificial Delta:Omicron mixes and all positive controls (cell culture isolates) sequenced during the study period (n=1084, including 892 pure cell culture isolates and 192 experimental mixes) a ROC curve was generated with the Youden index indicating a putative optimal point for this cutoff (**Fig S1A**). While the Youden index's best score corresponds to a minimum of 4 lineage-specific variants cutoff (TPR-FPR=0.952), the chosen cutoff of 6 while designing the study had a close score (TPR-FPR=0.932) and permitted the exclusion of false positives identified in the real dataset. Indeed, out of 7 samples which had 4 or 5 secondary lineage-specific mutations at first passage, only 1 sample retained 5 secondary lineage-specific mutations at second passage (**Fig S2**). Moreover, in the real dataset, among samples with a non-null secondary lineage-specific mutations count, 2143/2257 (94.9%) samples had a mutation count ranging from 1 to 5 as shown in **Fig S1B**, which was similar to the proportion of 454/477 (95,1%) found in the positive controls sequenced during the study period.

Impact of the cutoff value on different lineages detection

The number of defining mutations depends on the lineage with a median of 40 defining mutations per lineage, a minimum of 26 mutations for AY.9.2 and a maximum of 64 mutations for BA.2 (**Fig S3A**). For the main lineage definition, the 6 defining mutations threshold is very low compared to the number of defining mutations per lineage (**Fig S3A**). On the other hand, the 6 specific mutations threshold to find a secondary lineage limits our ability to detect co-infections between closely related lineages. Specifically, 12321/43472 (28%) Delta/Delta co-

infections with different lineages cannot be detected using the 6 specific mutation threshold (**Fig S3B**). At the time of writing, there was only one sublineage of BA.1 defined, BA.1.1, and co-infections between BA.1 and BA.1.1 would not be detected either as BA.1.1 has only 3 specific mutations in addition to the defining mutations of BA.1.

Supplementary Results

Impact of the cutoff value on the prevalence of natural co-infections

The number of samples with a secondary lineage identified in their first replicate depends on the cutoff value used to determine the secondary lineage (**Fig S7**). The chosen cutoff was 6 specific mutations, resulting in 0.29% of potentially co-infected samples (95% CI: 0.22%-0.37%, assuming a binomial distribution). Using only 1 specific mutation as the cutoff would identify a potential co-infection in 10.3% of the samples. In contrast, a cutoff between 3 and 17 specific mutations would not significantly change the estimated prevalence of co-infection (Fisher's p-value >0.05) (**Fig S7**). Excluding 1 specific mutation, the estimated prevalence varied between 0.542% (for a cutoff of 2 specific mutations) and 0.00935% (for a cutoff of 44 defining mutations).

Primer bias

Detection rates of Omicron-specific mutations were lower than detection rates of Delta-specific mutations, using both the co-variant list of mutations and the seqmet-db agnostic list of mutations specific to B.1.617.2 and BA.1 lineages. In mixes with 10% expected frequency of Omicron (90:10 mixes), most Omicron-specific mutations were missed because they did not reach 5% relative frequency (default threshold for variant calling used in seqmet) (**Table S3**). In addition, Omicron-specific mutations comprised in Artic V4 amplicons 76 (nt 22677- 23028) and 89 (26587-26956); or Midnight V1 and V2 amplicons 24 and 28 (nt 23544-24714 and 27808 – 28985) were missed in mixes with low frequency of Omicron sequenced with those

primers because of preferential amplification of Delta over Omicron in those regions (**Table S3**).

Importantly, amplification bias of previously listed primer sets led to chimeric SARS-CoV-2 genome sequences characterized by the presence of Delta-specific mutations in Omicron sequences for mixes with 10 to 30% Delta (**Fig S4, Fig S5**). The highest number of Delta-specific mutations found in these chimeric sequences were observed in mixes with 50% measured frequency of Delta, which were mixes with 20% expected frequency of Delta sequenced with Midnight primers, and mixes with 30% expected frequency of Delta sequenced with Artic primers. Primer sets responsible for much of the chimera were Midnight primer 28 (including the Delta-specific ORF8:118Del and N:D63G mutations) and Artic V4 primers 76 (including the Delta-specific S:L452R mutation) and 89 (including the Delta-specific M:I82T mutation).

Detection limit of the seqmet pipeline

To test the detection limit of our pipeline to detect co-infections in mixes with lower percentage of Omicron that may also lead to chimeric consensus sequences, we performed additional mixes with Delta:Omicron ratios of 1:99 and 5:95 that were sequenced in 10 replicates (**Fig S6** and **Table S4**). Chimeric sequences were defined as sequences with both Delta- and Omicron-specific mutations in the consensus. For the 5:95 mixes, chimeric sequences were found in 10/10, 9/10, 10/10 and 6/10 samples sequenced with Midnight V1, Midnight V2, Artic V4 and Artic V4.1, respectively. All the 5:95 mixes had a positive secondary lineage mutation ratio and were thus identified as co-infections. In contrast, for the 1:99 mixes, the seqmet pipeline identified a co-infection in 9/10, 9/10, 7/10 and 7/10 samples, while 7/10, 1/10, 10/10, and 0/10 were chimeric sequences with Midnight V1, Midnight V2, Artic V4 and Artic V4.1, respectively. Therefore, for mixes with only 1% of Omicron, our workflow was not able to detect the co-infection in all replicates, while one of the 1:99 mix sequenced with Midnight V1

was detected as not co-infected but led to chimeric sequences. This detection limit was expected as the threshold used for variant calling is 5%, therefore, in theory, only minor viruses above 5% should be detected. However, because of primer bias preferentially amplifying Delta over Omicron, in the 1:99 mixes, Delta had a measured relative abundance above 5%.

Investigation of samples with discordant secondary lineage mutation ratios in duplicate

Among 61 samples with positive secondary lineage mutation ratios in the first passage, 8 samples had null ratios in the second passage, thus no longer found to harbour co-infections. These samples were characterized by lower secondary lineage mutation ratios in the first replicate than confirmed co-infected samples (median ratio of 0.38 vs 0.78, Kruskal-Wallis p-value = 0.0083) (**Fig S8A**) and lower relative abundance of the minor lineage in the first replicate (median relative abundance of 9.5% vs 18%, Kruskal-Wallis p-value = 0.03) (**Fig S8 B**). The distribution of minor alleles along the genome in duplicate suggests that a contamination arose during the first sequencing process, either during RNA extraction, PCR amplification or library preparation (**Fig S9**). For ⅝ samples, minority variants from the secondary lineage were present all along the genome, suggesting that the contamination arose most likely during the RNA extraction. For ⅜ samples, minority variants were present only on 2 to 3 amplicons, suggesting contamination may have arisen after PCR amplification with a limited number of amplicons. Additionally, for 4 samples, as secondary lineage-specific mutation relative frequencies were ~5%, we cannot rule out that a co-infection could have been present, but allele frequencies were not reproducible around 5%.

Evaluation of uniform frequencies among co-infected samples for screening of recombinant events

To assess whether recombinants may be present in co-infected samples, the cumulative sum of allele frequencies were used. We considered that when two specific lineages are present in

different proportions, the cumulative variant allele frequencies of the specific defining-lineage positions should follow a linear distribution. In addition, we considered that the linear distribution of each lineage should have a 0 intercept. If a recombinant is present, we would observe a break in uniformity of specific positions frequencies and thus a default in linear regression and a deviation from 0 intercept. From these hypotheses, we computed the adjusted r-squared value from the regression model of each major or minor lineage identified in samples and the p-value considering a 0 intercept under null hypothesis. To note, we removed nucleotide positions known to be associated with Artic primer bias regardless of the version (amplicons 76 and 88-90).

Adjusted r-squared of linear models from mixes were all above 0.98 (0.988-0.999). According to these results (**Fig S13**), we considered co-infected samples with an r-squared adjusted score under 0.98. Three samples were detected with consistent results in duplicate sequencing (021228537801; 0212296567 and 722000801801). Additionally, 5 samples had an adjusted R-squared below 0.98, but the uniformity of either major or minor lineage-specific frequencies was disrupted by sporadic positions. The pattern of these 5 samples is likely due to sequencing competition, whereas recombinant patterns follow a disruption in frequency uniformity affecting several and consecutive positions on different amplicons. By combining a threshold of adjusted R squared below 0.98 and a p.value below 0.05 testing 0 intercept, 3 samples were rejected and therefore suspected to be recombinant. This statistical approach within our pipeline is interesting for warning about possible recombinants. Nonetheless, visual inspection remains essential, as well as other specific investigations such as phasing and culture approaches to prove the presence of a recombinant among viral sub-populations.

Investigation of possible recombinants

Three possible recombinants occurring in co-infected samples may have been detected in this study. Virus isolation in cell culture was possible only for one of these recombinants

(021228537801, R1), as the two others samples had unfortunately been inactivated in the originating laboratories after sampling.

For the sample with a BA.1/BA.2 co-infection (sample # 722000801801, R2), a BA.1-predominant region was defined from nt 0 to 6,512 where the relative frequencies of BA.1-specific mutations were above 70%, while relative frequencies of BA.2-specific mutations were around 6% (**Fig S14B**). From nt 8,393 frequencies were reversed and BA.2 was predominant except for two positions (nt 22,686 and nt 22,673). Thus, this sample could contain a BA.1-BA.2 recombinant at 58% relative abundance with a likely breakpoint located between nt 6,512 nt and 8,393. Due to low viral load (Ct = 30), we could not conduct more investigations for this sample. Of note, this low viral load may be associated with poor reproducibility regarding variant allele frequencies.

Two potential recombinants were identified among Delta/Omicron co-infections (samples # 021228537801, R1 and 021229656701, R3). For 021228537801 (R1), a AY.43-predominant region was defined from nt 0 to 11,332, while BA.1.1 was predominant from nt 15,240 to 29,909 except for one position in one replicate (nt 22,917) (**Fig S14A**). The high viral load of this sample (Ct=22) enabled further investigations. First, we tested a long-read sequencing approach based on amplification with Midnight V2 primers, a ligation-based library preparation (PCR Barcoding Kit without fragmentation, Oxford Nanopore Technologies, ONT) and a sequencing on GridION platform with Q10 chemistry and FLO-MIN106 flow cell (ONT). Despite a read length of 1200 pb, no sufficient number of reads overlapping specific AY.43 and BA.1.1-specific mutations could be produced. This approach was therefore not contributive to specify the position of the breakpoint. Then, we tried to isolate the potential recombinant using viral cell culture Vero E6 TMPRSS2 cells with two successive passages (P1 and P2). WGS with Artic V4.1 primers was then performed on the two culture supernatants. A decrease of mixed populations throughout the passages was noticed (**Fig S14A**). In the P2 viral isolate, the

frequencies of AY.43-specific mutations reached 100% and BA.1.1 specific mutations were no longer detected from nt 0 to 15,240. From nt 21,762, AY.43-specific mutations were no longer detected and frequencies of BA.1.1-specific mutations reached 100%. From nt 15,240 to 19,220, both AY.43 and BA.1.1-specific mutations were still detected with median frequencies of 35% and 65%, respectively. These results suggest that two potential recombinants have been selected after the two passages in cell culture, the main one with a potential breakpoint around nt 15,000 and a final relative abundance of 65% and the minor one with a potential breakpoint around nt 20,000 and a final relative abundance of 35%. To explore this, the P2 viral isolate processed with Artic V4.1 primer was analyzed with a different bioinformatics pipeline (https://github.com/connor-lab/ncov2019-artic-nf). P2 was also processed with a metagenomics (mNGS) method based on Revelo RNA-Seq High Sensitivity kit (Tecan, Männedorf, Switzerland), Illumina sequencing and mapping-based bioinformatics analyses (seqmet pipeline, https://github.com/genepii/seqmet). Both approaches showed similar results to those obtained with V4.1 primer followed by seqmet analyses that ruled out sequencing artefacts in this region of the genome (**Fig S15**). Of note, the consensus sequence generated from the second passage was unclassified with Pango v.4.1.1, suggesting that these recombinants have not been significantly detected.

Finally, the investigation of 021229656701 (R3) revealed a potential recombinant at 13% relative abundance since an increase and a decrease of 13% were found respectively for the allele frequencies of specific mutations defining the minor and the major lineage after the position 23,604 and from 23,948, the two positions defining the suspected region for the recombinant breakpoint (**Fig S14C**). It is worth noting that contrary to the other recombinants discussed above, specific mutations of the minor lineage did not reach 50% frequency. The consensus sequence was thus not impacted.

Figure S1

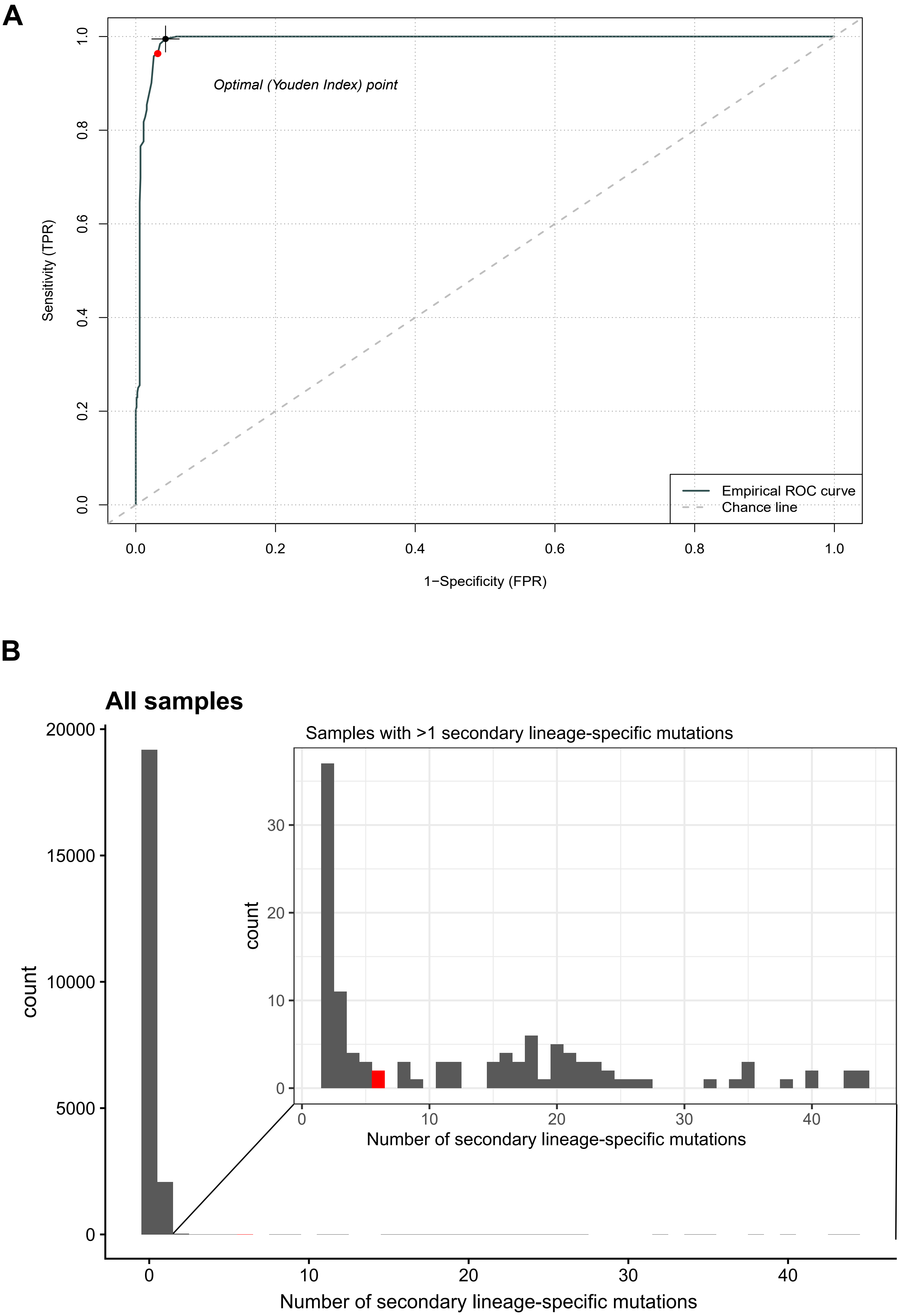

Fig S1: Determination of the optimal cutoff value for detecting the secondary lineage. A. Empirical ROC curve of the coinfection detection algorithm depending on the positivity cutoff chosen for Delta:Omicron mixes and all positive controls (cell culture isolates). Points depict the calculated optimal Youden index in black and the chosen cutoff in red. **B**. Distribution of the number of specific mutations in all sequenced samples (n=21,387 samples). The chosen cutoff (6 specific mutations) is in red.

Figure S2

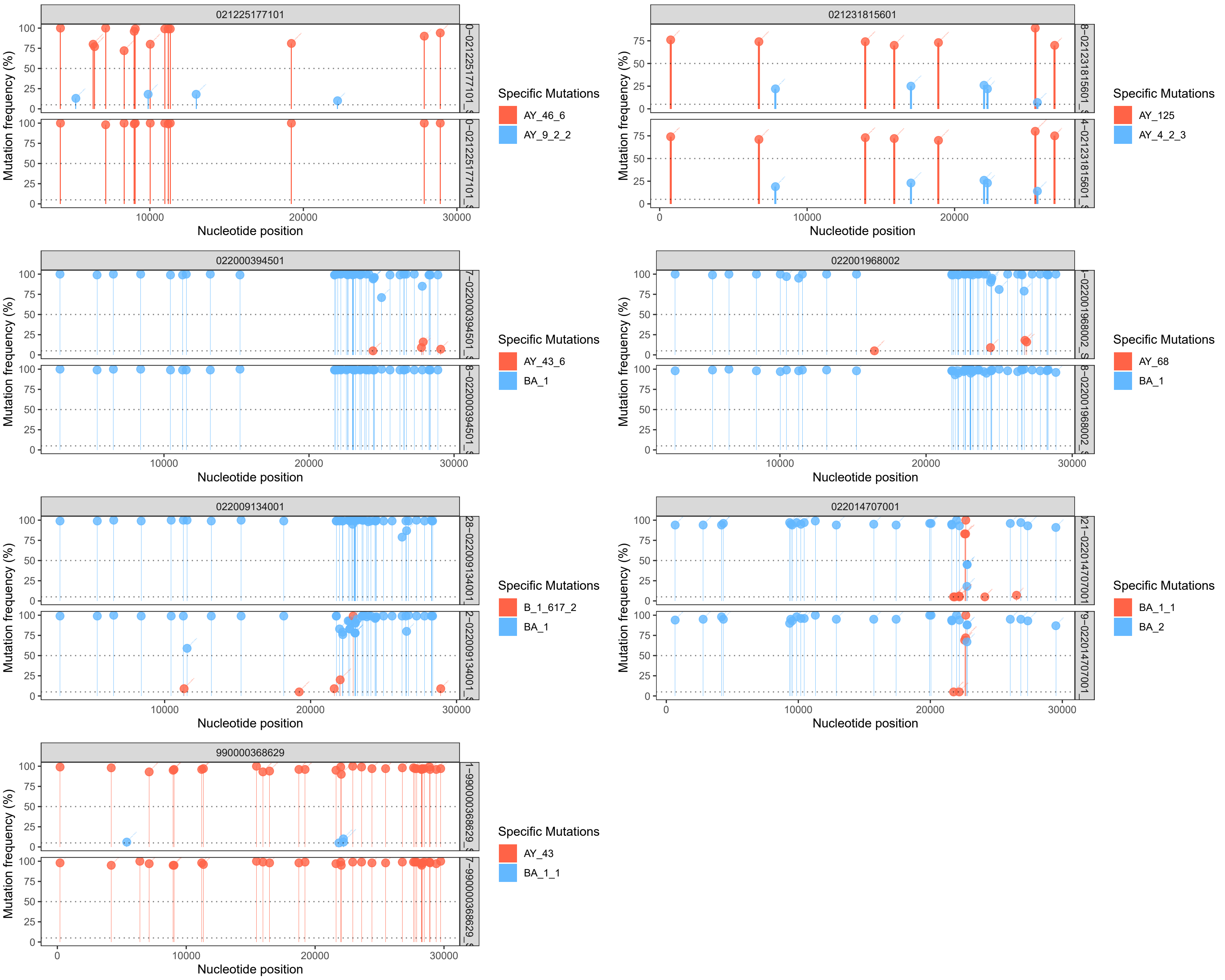

Figure S2: Representation of variant allele frequency of main- and secondary-specific mutations for 7 refuted co-infections based on the number of secondary lineage-specific mutations not reaching the 6 mutations cutoff. All 7 samples had 4 or 5 secondary lineage-specific mutations at first passage, but only 1 sample retained 5 secondary lineage-specific mutations at second passage. The list of specific mutations is based on the seqmet lineage mutation database. Main and secondary lineages identified by seqmet are represented by different colors as indicated in each legend panel. Shared mutations between main and secondary lineages were excluded. Horizontal lines at 50% show which mutations are called in the consensus sequence based on the majority rule.

Figure S3

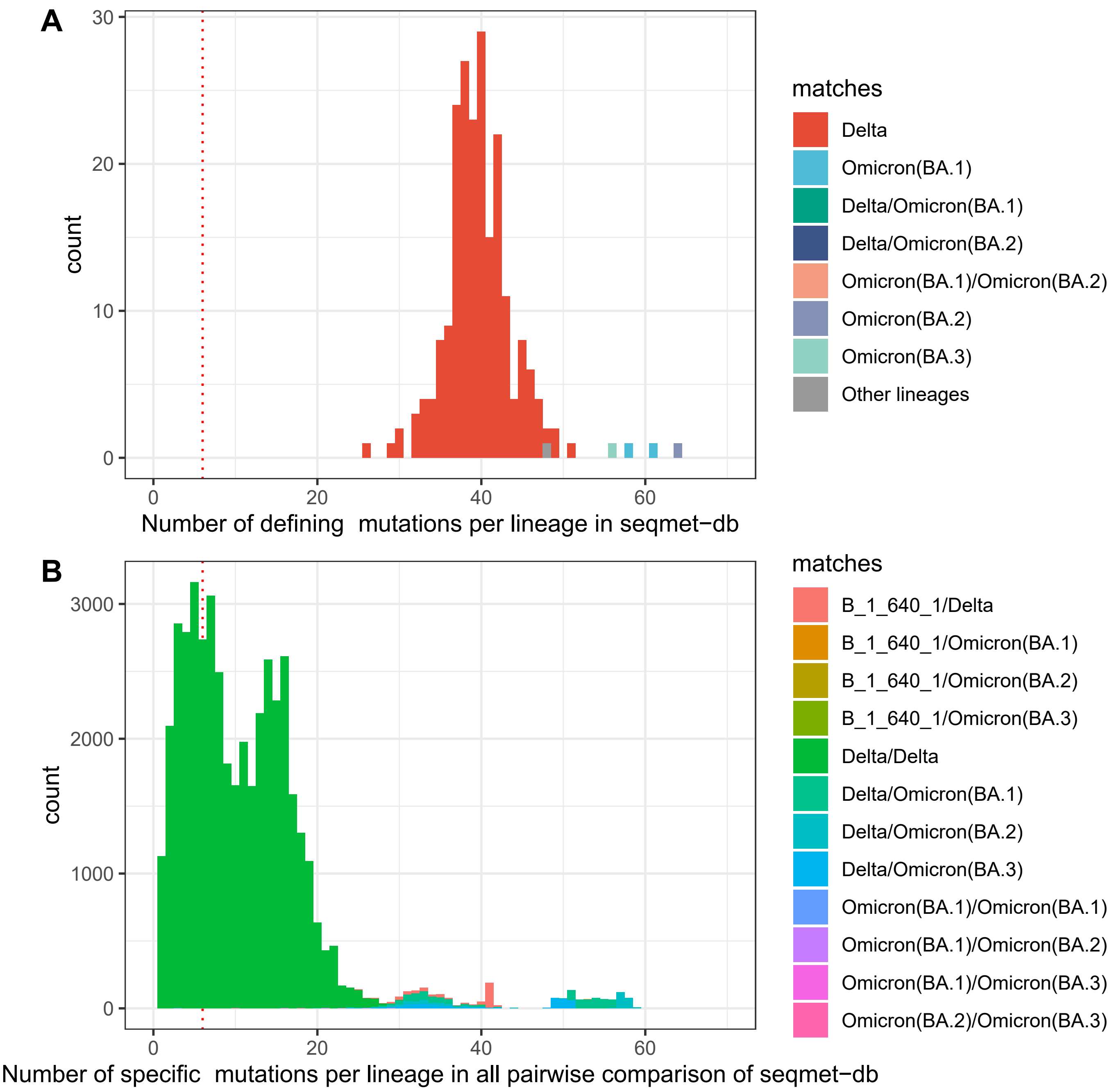

Fig S3: Agnostic list of defining and specific mutations per lineage. A. Distribution of defining mutations for each lineage in seqmet-db. **B**. Pairwise comparison of all lineages of the seqmet database to determine the distribution of specific mutations. Vertical lines at 6 show the 6 mutations cutoff used in the present study to identify main and secondary lineages.

Figure S4

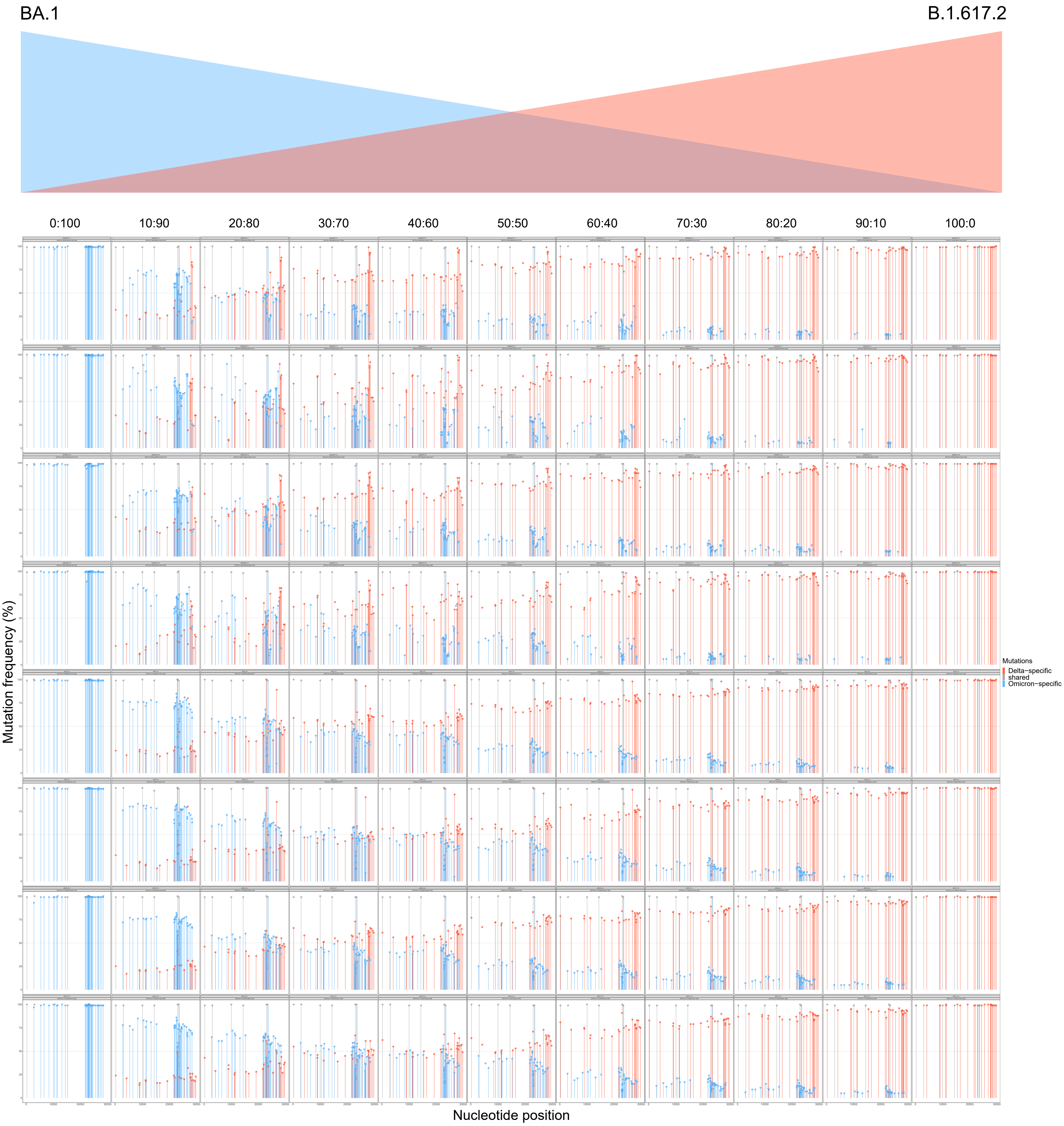

Figure S4: Representation of variant allele frequency of Delta- and Omicron-specific mutations for Delta:Omicron mixes. The list of specific mutations is based on co-variant.org. Delta-specific mutations are depicted in red, Omicron-specific mutations in blue and shared mutations are in grey. Horizontal lines at 50% show which mutations are called in the consensus sequence based on the majority rule.

Figure S5

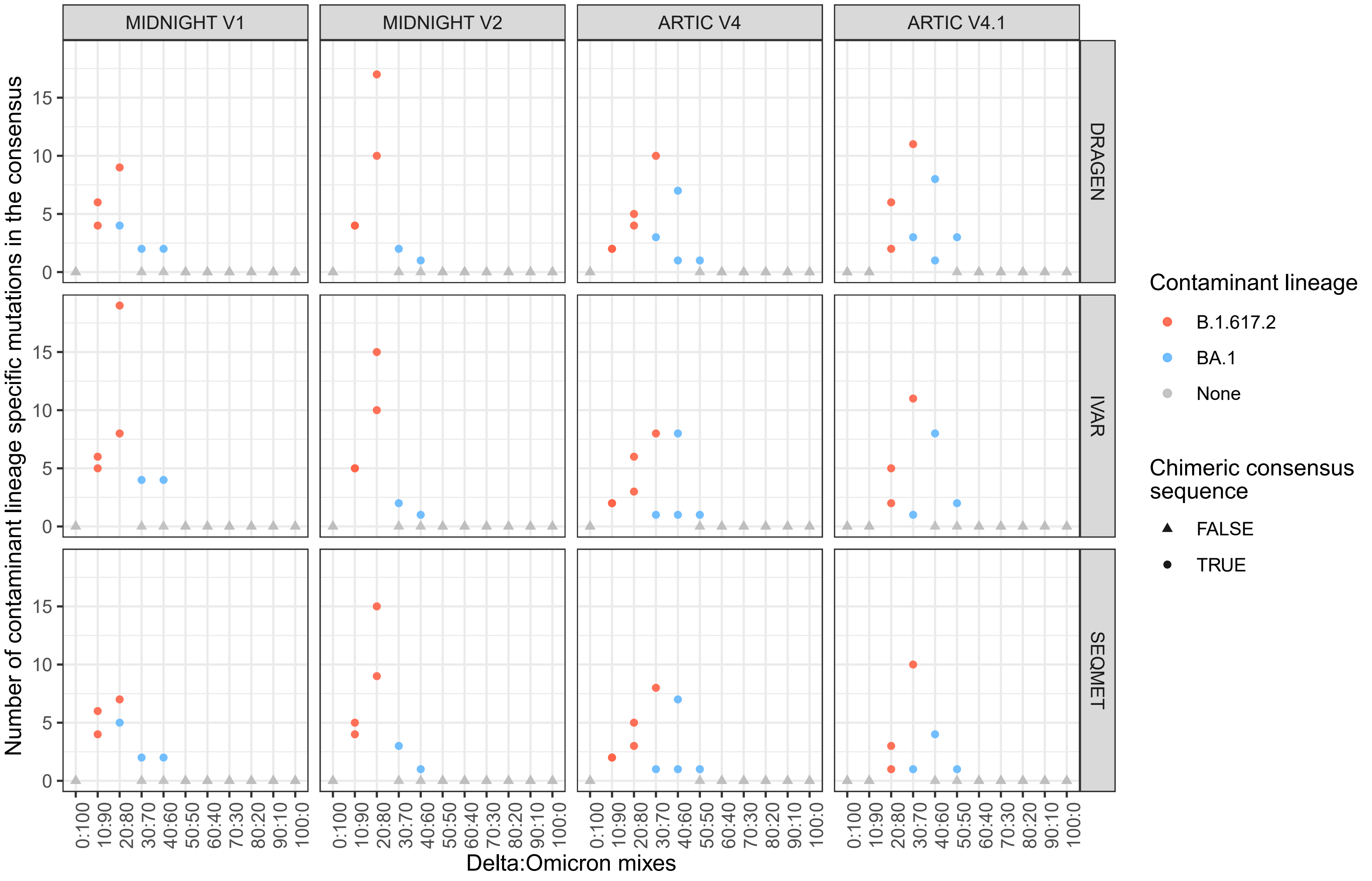

Fig S5: Chimerism in consensus sequences of Delta:Omicron mixes detected with different bioinformatic pipelines. The contaminant lineage was defined as the lineage with the lowest number of specific mutations within the consensus sequence. The list of specific mutations was based on covariants.org. The number of specific mutations from the contaminant lineage is used as a proxy to quantify chimerism in each sequence. Results from seqmet pipeline (used in the present study) were compared with two commonly used bioinformatic pipelines: DRAGEN and IVAR (https://github.com/connor-lab/ncov2019-artic-nf).

Figure S6

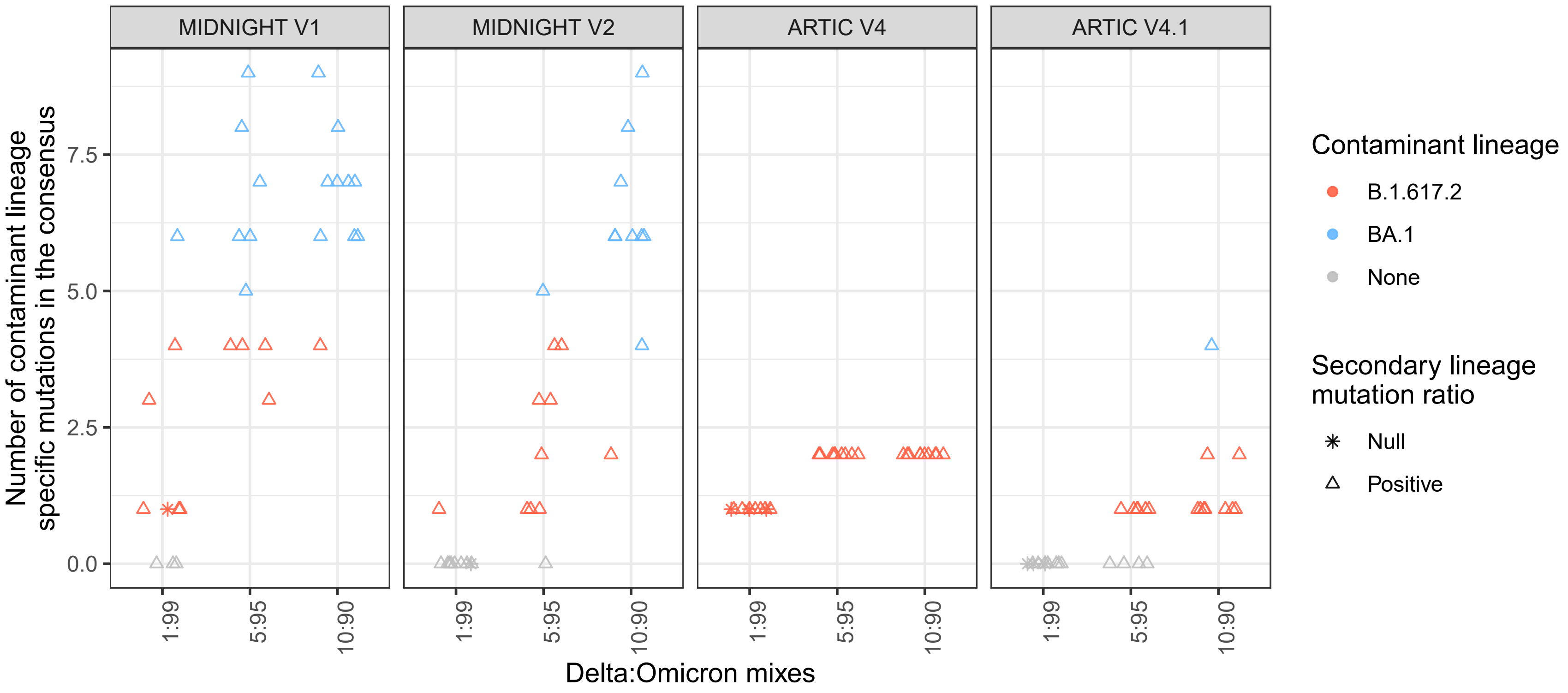

Fig S6: Chimerism in consensus sequences of Delta:Omicron mixes detected in additional Delta:Omicron mixes with ratios of 1:99, 5:95 and 10:90. The contaminant lineage was defined as the lineage with the lowest number of specific mutations within the consensus sequence. The list of specific mutations is based on the seqmet lineage mutation database. The number of specific mutations from the contaminant lineage is used as a proxy to quantify chimerism in each sequence. Samples identified as co-infected using seqmet (i.e. a positive secondary lineage mutation ratio) are depicted as triangles, while samples identified as pure samples (i.e. null secondary lineage mutation ratio) are depicted as stars.

Figure S7

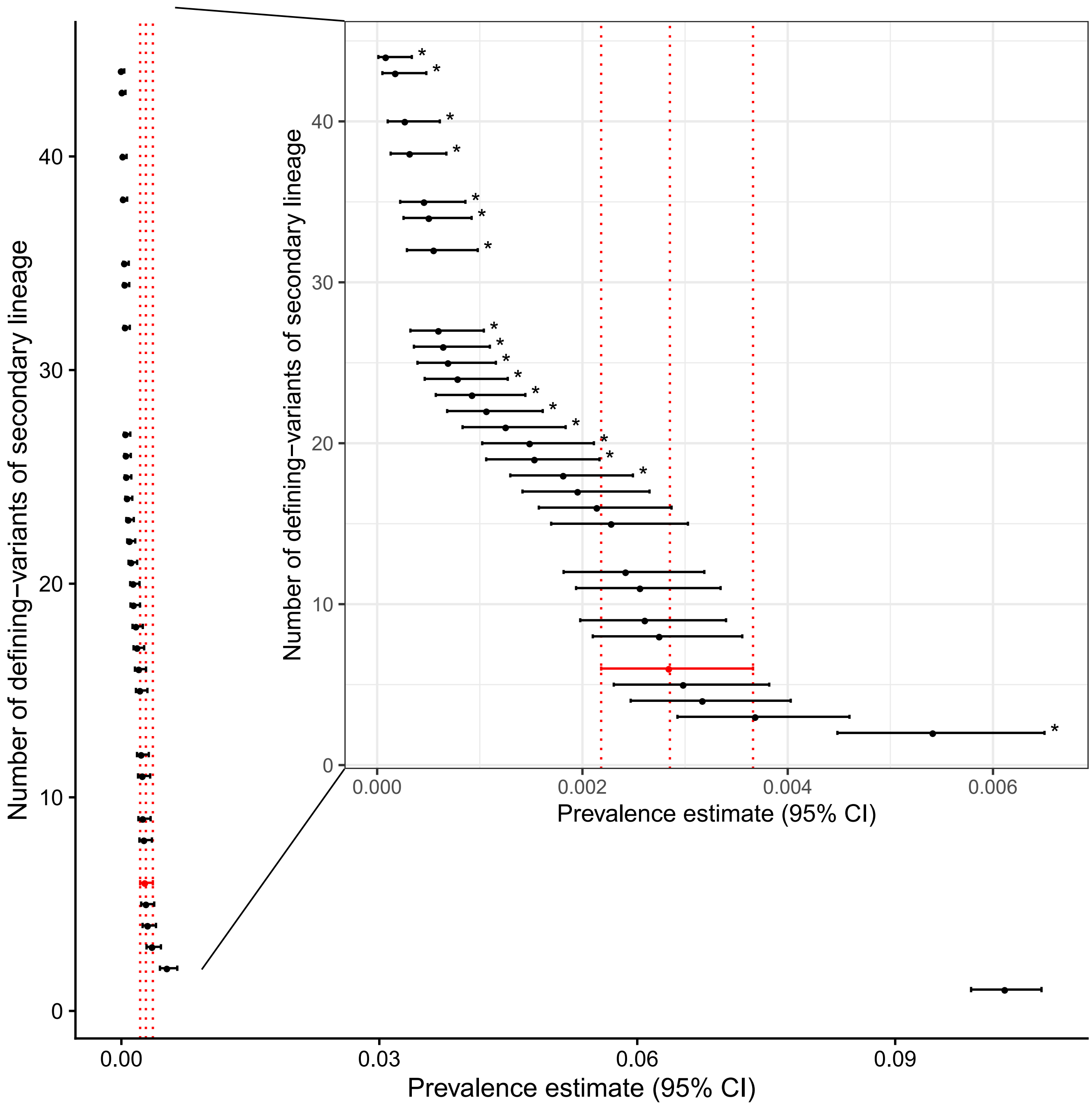

Figure S7: Impact of different specific mutation cutoffs on the prevalence of natural co-infections in all sequenced samples (n=21,387 samples). Data are presented as prevalence estimate with error bars representing the 95% CI assuming a binomial distribution. The chosen cutoff is in red, with red dotted lines representing 95% CI assuming a binomial distribution. In the inset, stars represent significantly different prevalence estimates compared with the chosen cutoff (two-sided Fisher test p-value<0.05). P-values from top to bottom of the inset are : <10-5, <10-5, <10-5, <10-5, <10-5, <10-5, <10-5, <10-5, <10-5, <10-5, <10-5, 0.00001, 0.00004, 0.00037, 0.00343, 0.00503, 0.03499, 0.07527, 0.17510, 0.29361, 0.45125, 0.64221, 0.71132, 0.92721, 1.00000, 0.85791, 0.59693, 0.14986, and 0.00004.

Figure S8

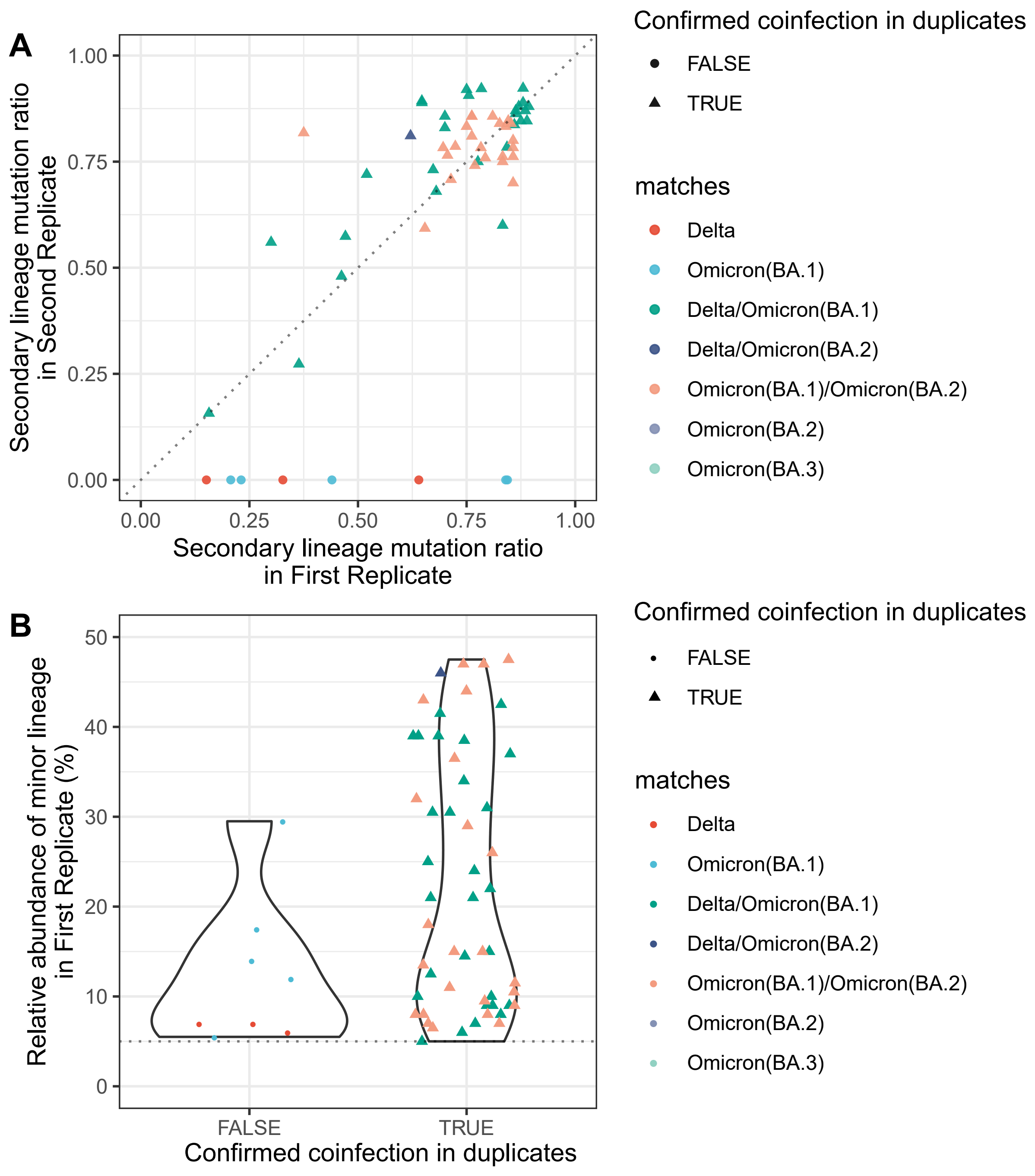

Figure S8: Reproducibility of seqmet co-infection detection scores among the 61 samples sequenced in duplicate. In both panels, co-infections between Delta/BA.1 are indicated in green, Delta/BA.2 in dark blue, and BA.1/BA.2 in salmon. **A.** Correlation between secondary lineage mutation ratios in first and second replicates. Samples found to harbor a coinfection (i.e. which have positive secondary lineage mutation ratios in duplicate) are depicted as triangles, while samples with discordant secondary lineage mutation ratios are depicted as dots. **B.** Violin plots showing the distribution of relative abundance of the minor lineage in refuted and confirmed natural co-infection. Shapes indicate whether the minor lineage is identified as the secondary or main lineage in duplicate, or a potential discordance between duplicates. The horizontal line at 5% shows the allele frequency threshold used to call variants in seqmet.

Figure S9

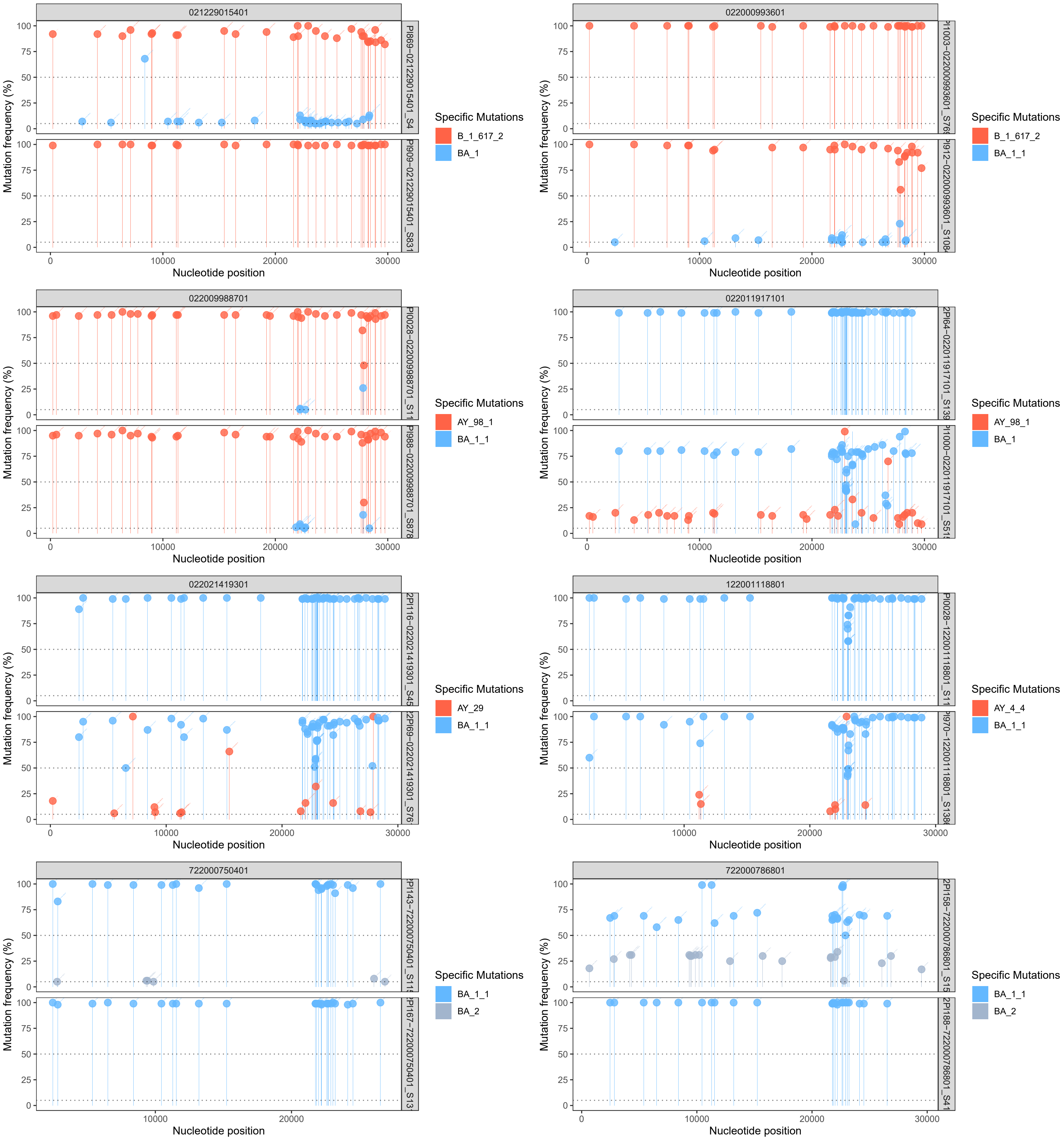

Figure S9: Representation of variant allele frequency of main- and secondary-specific mutations for 8 refuted co-infections based on discordant secondary lineage mutation ratios in duplicate sequencing. The list of specific mutations is based on the seqmet lineage mutation database. Main and secondary lineages identified by seqmet are represented by different colors as indicated in each legend panel. Shared mutations between main and secondary lineages were excluded.

Figure S10

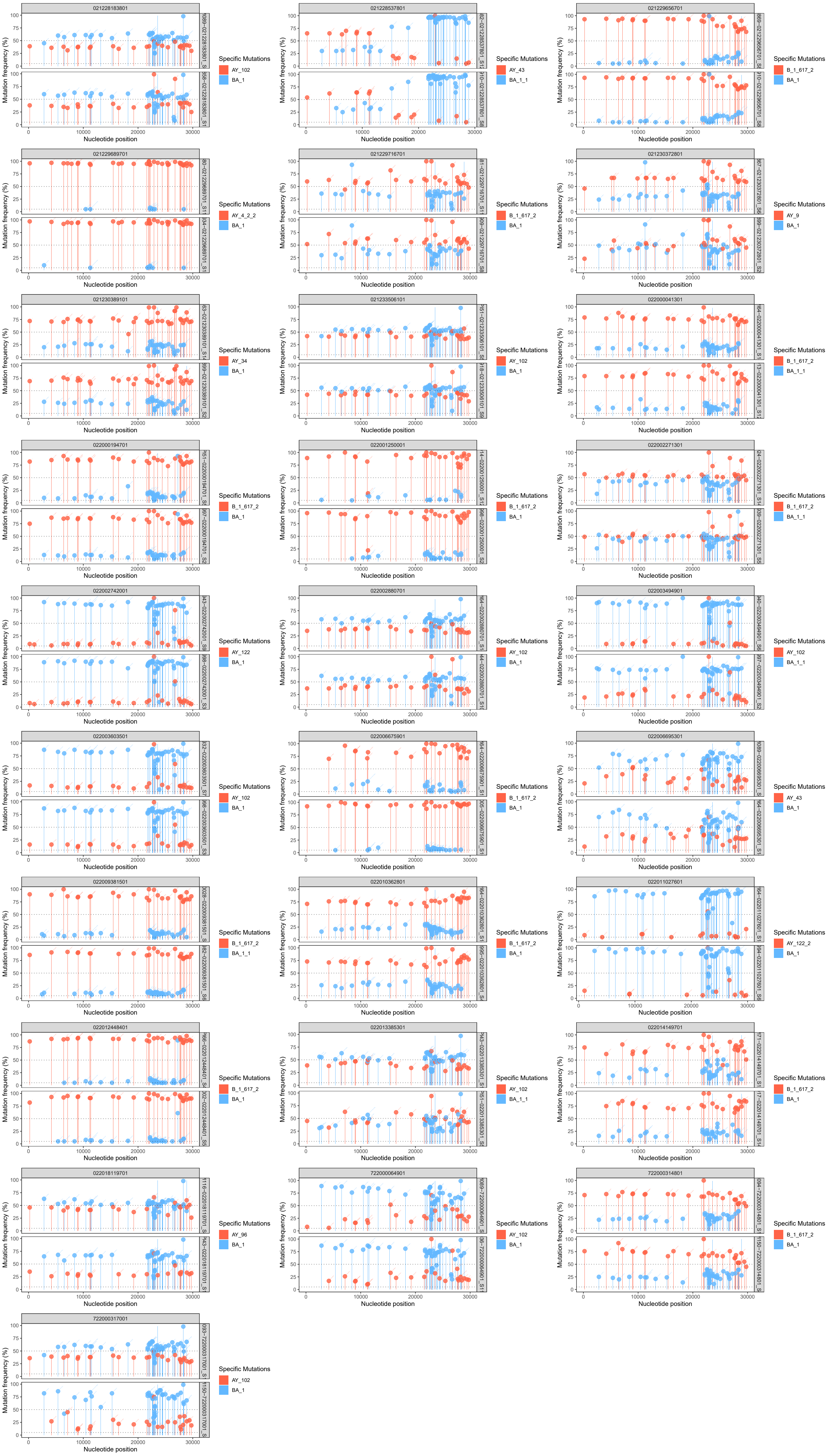
021228183801
Specific Mutations
AY_102
BA_1
021228537801
Specific Mutations
AY_43
BA_1_1
021229656701
Specific Mutations
B_1_617_2
BA_1
021229689701
Specific Mutations
AY_4_2_2
BA_1
021229716701
Specific Mutations
B_1_617_2
BA_1
021230372801
Specific Mutations
AY_9
BA_1
021230389101
Specific Mutations
AY_34
BA_1
021233506101
Specific Mutations
AY_102
BA_1
022000041301
Specific Mutations
B_1_617_2
BA_1_1
022000194701
Specific Mutations
B_1_617_2
BA_1
022001250001
Specific Mutations
B_1_617_2
BA_1
022002271301
Specific Mutations
B_1_617_2
BA_1_1
022002742001
Specific Mutations
AY_122
BA_1
022002880701
Specific Mutations
AY_102
BA_1
022003494901
Specific Mutations
AY_102
BA_1_1
022003603501
Specific Mutations
AY_102
BA_1
022006675901
Specific Mutations
B_1_617_2
BA_1
022006695301
Specific Mutations
AY_43
BA_1
022009381501
Specific Mutations
B_1_617_2
BA_1_1
022010362801
Specific Mutations
B_1_617_2
BA_1
022011027601
Specific Mutations
AY_122_2
BA_1
022012448401
Specific Mutations
B_1_617_2
BA_1
022013385301
Specific Mutations
AY_102
BA_1_1
022014149701
Specific Mutations
B_1_617_2
BA_1
022018119701
Specific Mutations
AY_96
BA_1
722000064901
Specific Mutations
AY_102
BA_1
722000314801
Specific Mutations
B_1_617_2
BA_1
722000317001
Specific Mutations
AY_102
BA_1
Mutation frequency (%)
Nucleotide position

Figure S10: Representation of variant allele frequency of Delta- and BA1-specific mutations for 28 samples detected as Delta/Omicron (BA.1) co-infections in duplicate sequencing. The list of specific mutations is based on the seqmet lineage mutation database. Main and secondary lineages identified by seqmet are indicated in each legend panel, with Delta-specific mutations are depicted in red, and BA.1-specific mutations in blue. Shared Delta- and BA.1-defining polymorphisms were excluded. Horizontal lines at 50% show which mutations are called in the consensus sequence based on the majority rule.

Figure S11

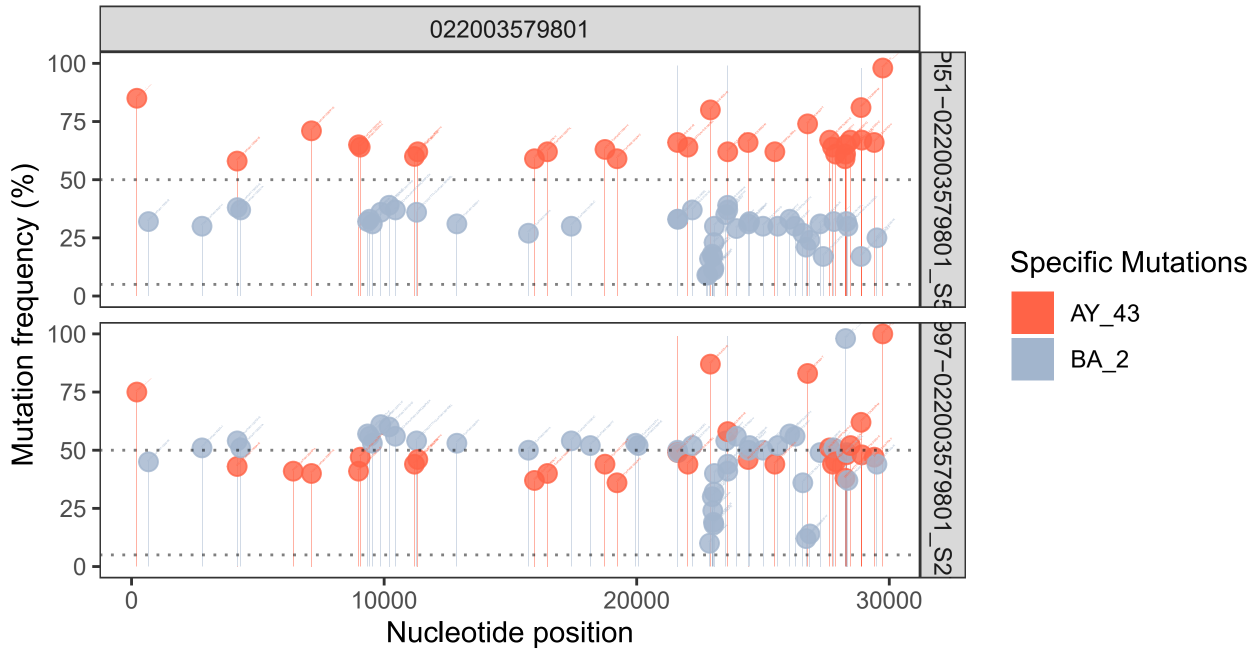

Figure S11: Representation of variant allele frequency of Delta- and BA.2-specific mutations for 1 **sample detected as Delta/Omicron (BA.2) co-infections in duplicate sequencing.** The list of specific mutations is based on the seqmet lineage mutation database. Main and secondary lineages identified by seqmet are indicated in each legend panel, with Delta-specific mutations are depicted in red, BA.2-specific mutations in blue grey. Shared Delta- and BA.2-defining polymorphisms were excluded. Horizontal lines at 50% show which mutations are called in the consensus sequence based on the majority rule.

Figure S12

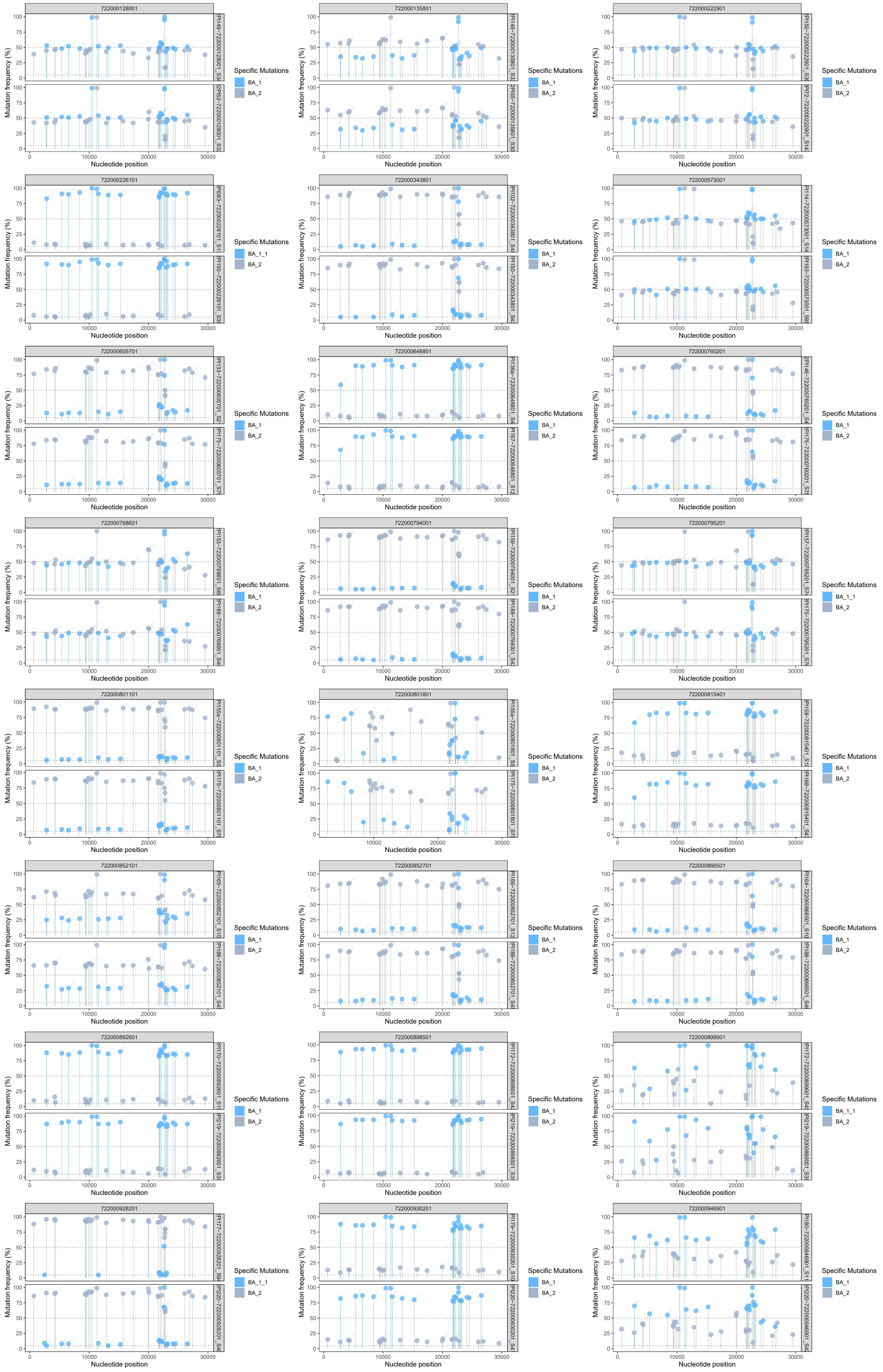

722000128001
722000135801
722000222901
722000226101
722000343801
722000573001
722000605701
722000648801
722000760201
722000768601
722000794001
722000795201
722000801101
722000801801
722000815401
722000852101
722000852701
722000866501
722000892601
722000898501
722000899001
722000928201
722000930201
722000946901
Mutation frequency (%)
Nucleotide position
0
10000
20000
30000
100
75
50
25
0
Specific Mutations
BA_1
BA_1_1
BA_2

Figure S12: Representation of variant allele frequency of BA.1- and BA.2-specific mutations for 24 samples detected as BA.1/BA.2 co-infections in duplicate sequencing. The list of specific mutations is based on the seqmet lineage mutation database. Main and secondary lineages identified by seqmet are indicated in each legend panel, with BA.1-specific mutations are depicted in blue, BA.2-specific mutations in blue grey. Shared BA.1- and BA.2-defining polymorphisms were excluded. Horizontal lines at 50% show which mutations are called in the consensus sequence based on the majority rule.

Figure S13

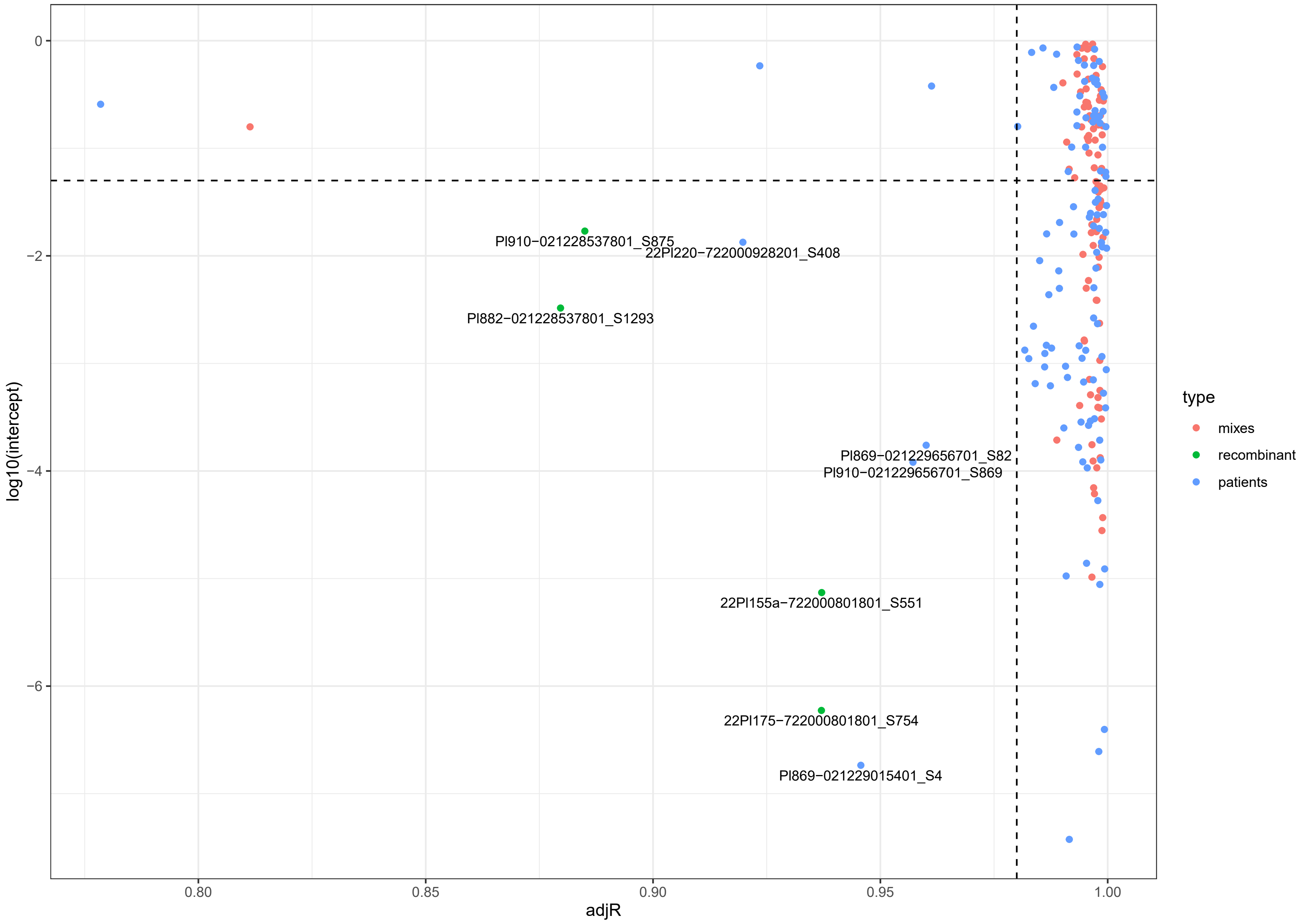

Figure S13: **Distribution of co-infected samples according to statistical parameters assessing uniformity of specific allele frequencies of minor lineages.** Co-infected samples, including mixes (red), patients (blue) and the two recombinants (green) are represented. The y-axis represents the negative $\log_{10}$ (p-value) testing 0 intercept (two-sided Student test) from the cumulative sum of specific minor lineage frequencies. The x-axis represents the adjusted R-squared of the regression line fitted from the linear model of the cumulative sum of specific minor lineage frequencies. Dashed lines show the two thresholds: 0.98 for adjusted-squared and -1.3 for $\log_{10}$ (p-value) which represents a p-value below 0.05.

Figure S14

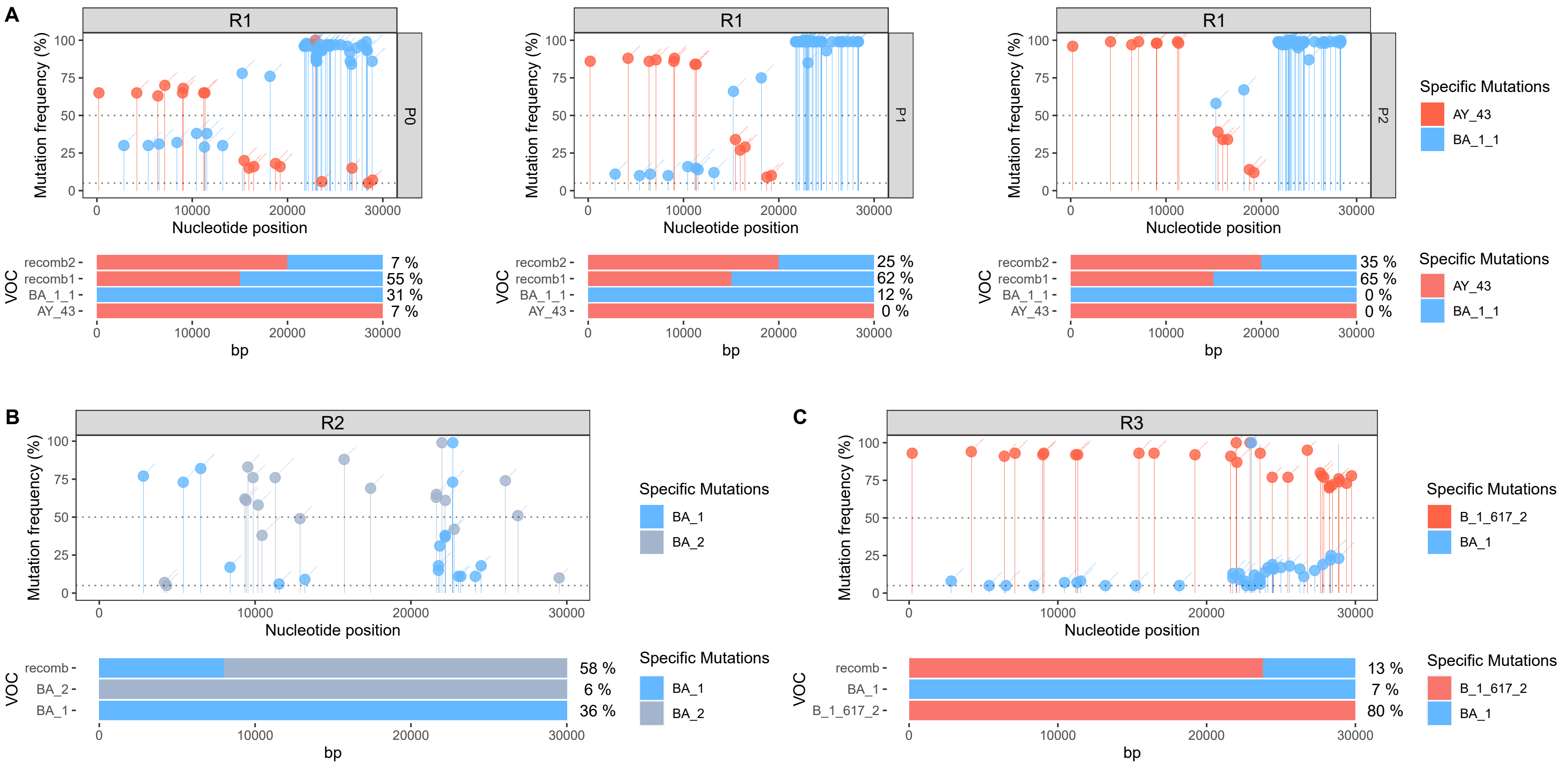

Figure S14: Representation of variant allele frequency for the potential recombinants and their hypothesized relative abundance within each co-infected sample. The list of specific mutations is based on the seqmet lineage mutation database. Main and secondary lineages identified by seqmet are represented by different colors as indicated in each legend panel. Shared mutations between main and secondary lineages were excluded. Horizontal lines at 50% show which mutations are called in the consensus sequence based on the majority rule. Horizontal bar plots below each panel depict the estimation of relative abundance of each viral population within each sample. Breakpoints were visually determined based on change in variant allele frequencies, and median of variant allele frequencies before and after the breakpoints were used to estimate the relative abundance of the co-infecting lineages and potential recombinants. Each panel depicts a sample: A. 021228537801 (R1) with breakpoints = 15,000 and 20,000. P0: the initial NPS sample, P1: primary cell culture isolate and P2: viral isolate after the second serial cell culture passage; B. 722000801801 (R2) with breakpoint = 8,000; and C. 021229656701 (R3) with breakpoint = 23,800.

Figure S15

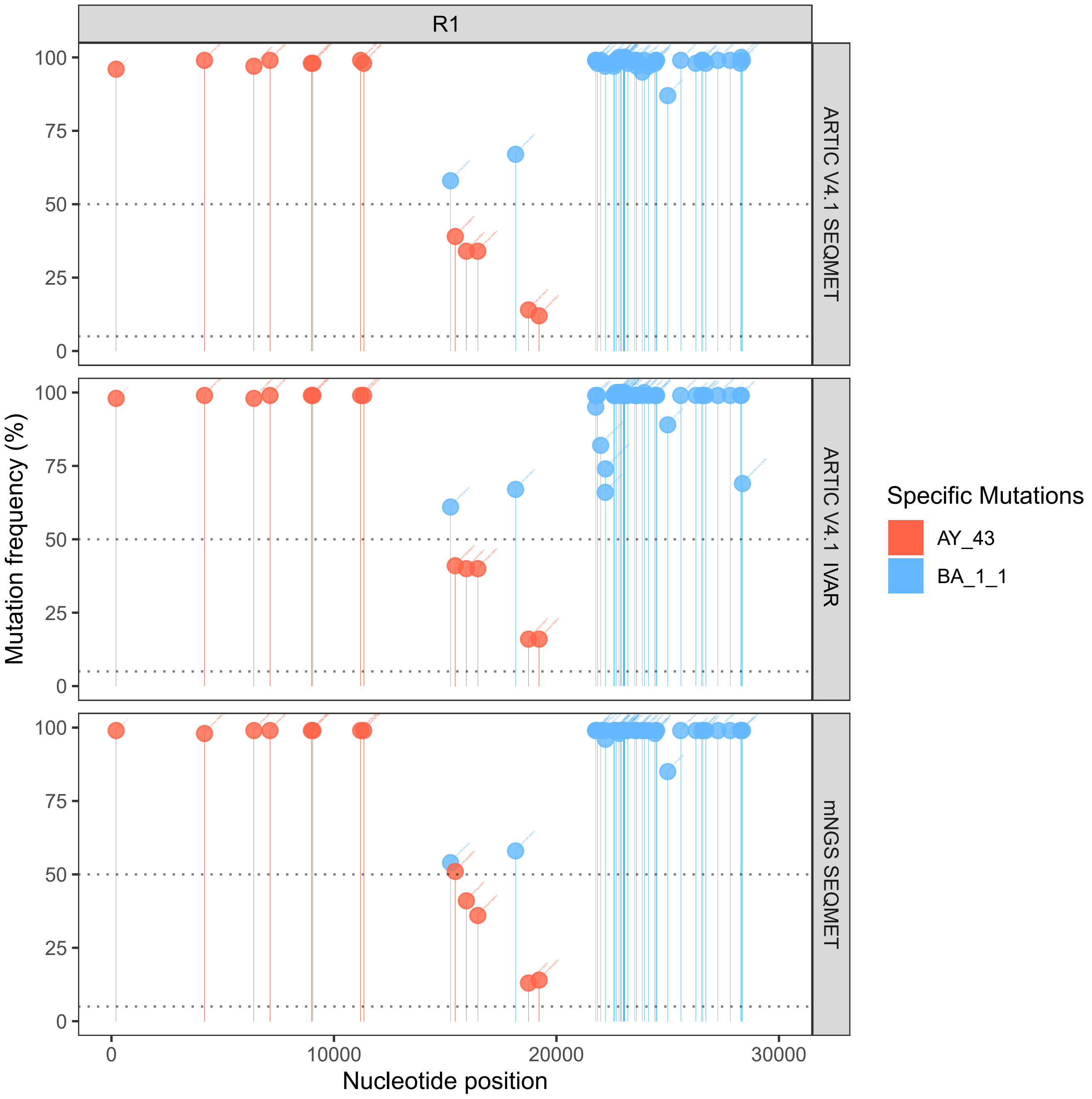

Figure S15. Representation of variant allele frequency of AY.43- and BA.1-specific mutations for the viral isolate after the second serial cell culture passage (P2) of sample #021228537801 (R1). To rule-out potential experimental or bioinformatics artifact, the P2 was sequenced with ARTIC V4.1 primers and metagenomics (mNGS), and analyzed with both seqmet and IVAR pipelines. The list of specific mutations is based on the seqmet lineage mutation database. Main and secondary lineages identified by seqmet are indicated in each legend panel, with **AY.43**-specific mutations are depicted in red, and BA.1-specific mutations in blue. Shared Delta- and BA.1-defining polymorphisms were excluded. Horizontal lines at 50% show which mutations are called in the consensus sequence based on the majority rule.

Table S1

Tools	Versions
abra2	2.24
bcftools	1.13
bedtools	2.30.0
bowtie2	2.4.2
constellations	v0.1.2
cutadapt	3.3
freebayes	1.3.5
htslib	1.13
minimap2	2.18
nextclade	1.10.2
pango-designation	v1.2.126
pangoLEARN	2022-01-20
pangolin	3.1.20
picard	2.25.0
samtools	1.13
scorpio	v0.3.16
seqtk	1.3
vt	0.57721

Table S1: Versions of tools used in the seqmet bio-informatic pipeline.

Supplementary Data 1: Sequencing results for Delta:Omicron mixes. Analysis results of the vcf based on the covariant list of Delta- and Omicron-specific mutations are compared with results of our unbiased co-infection detection script.

Supplementary Data 2: Allele frequencies in Delta:Omicron mixes for each Delta- and Omicron-specific positions defined in covariants and/or seqmet-db (as indicated in the source column). A white to back gradient color indicates 0 to 100% allele frequency. Alleles are ordered based on their frequency profiles. Non covered positions (NA) are highlighted in yellow.

Supplementary Data 3: Sequencing results for additional 1:99, 5:95 and 10:90 Delta:Omicron mixes.
