# Supplementary material for: Detection and prevalence of SARS-CoV-2 co-infections during the Omicron variant circulation in France
Source: Nat Commun. 2022 Oct 23;13:6316. doi: 10.1038/s41467-022-33910-9 (PMC9588762; doi:10.1038/s41467-022-33910-9)
Supplement: Supplementary file 3 — Description of Additional Supplementary Files [file 41467_2022_33910_MOESM3_ESM.pdf]

Description of Additional Supplementary Files

File Name: Supplementary Data 1
Description: **Sequencing results for Delta:Omicron mixes.** Analysis results of the vcf based on the covariant list of Delta- and Omicron-specific mutations are compared with results of our unbiased co-infection detection script.

File Name: Supplementary Data 2
Description: **Allele frequencies in Delta:Omicron mixes for each Delta- and Omicron-specific positions defined in covariants and/or seqmet-db** (as indicated in the source column). A white to back gradient color indicates 0 to 100% allele frequency. Alleles are ordered based on their frequency profiles. Non covered positions (NA) are highlighted in yellow.

File Name: Supplementary Data 3
Description: **Sequencing results for additional 1:99, 5:95 and 10:90 Delta:Omicron mixes.**
