# Supplementary material for: Detection and prevalence of SARS-CoV-2 co-infections during the Omicron variant circulation in France
Source: Nat Commun. 2022 Oct 23;13:6316. doi: 10.1038/s41467-022-33910-9 (PMC9588762; doi:10.1038/s41467-022-33910-9)
Supplement: Supplementary file 7 — Reporting Summary [file 41467_2022_33910_MOESM7_ESM.pdf]

nature portfolio

Corresponding author(s): Laurence Josset

Last updated by author(s): Sep 30, 2022

Reporting Summary

Nature Portfolio wishes to improve the reproducibility of the work that we publish. This form provides structure for consistency and transparency in reporting. For further information on Nature Portfolio policies, see our Editorial Policies and the Editorial Policy Checklist.

Statistics

For all statistical analyses, confirm that the following items are present in the figure legend, table legend, main text, or Methods section.

n/a	Confirmed	
☐	☒	The exact sample size (n) for each experimental group/condition, given as a discrete number and unit of measurement
☐	☒	A statement on whether measurements were taken from distinct samples or whether the same sample was measured repeatedly
☐	☒	The statistical test(s) used AND whether they are one- or two-sided<br>*Only common tests should be described solely by name; describe more complex techniques in the Methods section.*
☐	☒	A description of all covariates tested
☒	☐	A description of any assumptions or corrections, such as tests of normality and adjustment for multiple comparisons
☐	☒	A full description of the statistical parameters including central tendency (e.g. means) or other basic estimates (e.g. regression coefficient) AND variation (e.g. standard deviation) or associated estimates of uncertainty (e.g. confidence intervals)
☐	☒	For null hypothesis testing, the test statistic (e.g. F, t, r) with confidence intervals, effect sizes, degrees of freedom and P value noted<br>*Give P values as exact values whenever suitable.*
☒	☐	For Bayesian analysis, information on the choice of priors and Markov chain Monte Carlo settings
☒	☐	For hierarchical and complex designs, identification of the appropriate level for tests and full reporting of outcomes
☒	☐	Estimates of effect sizes (e.g. Cohen's d, Pearson's r), indicating how they were calculated

Our web collection on statistics for biologists contains articles on many of the points above.

Software and code

Policy information about availability of computer code

Data collection	No software was used for data collection
Data analysis	The seqmet bioinformatic pipeline is publicly available at https://github.com/genepii/seqmet. The software versions are provided in Table S1 The '58a2c4d28288b54cba425225bdaa9a0d642048ca' commit of seqmet should be used to reproduce the results presented herein. In addition, all data and R scripts used to generate the results are publicly available at https://github.com/genepii/seqmet/tree/main/script/article. Statistical analyses were conducted using R software, version 4.0.5 (R Foundation for Statistical Computing)

For manuscripts utilizing custom algorithms or software that are central to the research but not yet described in published literature, software must be made available to editors and reviewers. We strongly encourage code deposition in a community repository (e.g. GitHub). See the Nature Portfolio guidelines for submitting code & software for further information.

Data

Policy information about availability of data

All manuscripts must include a data availability statement. This statement should provide the following information, where applicable:

- Accession codes, unique identifiers, or web links for publicly available datasets
- A description of any restrictions on data availability
- For clinical datasets or third party data, please ensure that the statement adheres to our policy

The GISAID accession numbers of the Delta and Omicron virus isolates used for experimental mixes are EPI_ISL_11171170 and EPI_ISL_11171169, respectively. Sequencing data of the Delta:Omicron mixes were deposited on the SRA database under accession PRJNA817870 and PRJNA853723, and dehosted sequencing data of NPS with co-infections were deposited under accession PRJNA817806.

Field-specific reporting

Please select the one below that is the best fit for your research. If you are not sure, read the appropriate sections before making your selection.

☒ Life sciences ☐ Behavioural & social sciences ☐ Ecological, evolutionary & environmental sciences

For a reference copy of the document with all sections, see nature.com/documents/nr-reporting-summary-flat.pdf

Life sciences study design

All studies must disclose on these points even when the disclosure is negative.

Sample size	The sample size was not predetermined in this study. The samples sequenced at the National Reference Center (NRC) of Respiratory Viruses of Hospices Civils de Lyon (HCL) selected for this study were i) all samples from systematic sequencing of hospitalized patients in the Lyon area (university hospital of Lyon, HCL) and from HCL health care workers; ii) samples from random sequencing performed during the weekly Flash survey conducted by the EMERGEN consortium (French consortium for the genomic surveillance of emerging pathogens). The Flash surveys are nationwide surveys where all private and public diagnostic laboratories in France are asked to provide to the NRC and other sequencing centers a fraction of positive samples from one day per week ranging from 25% to 100% according to the number of positive cases detected at the national level.
Data exclusions	No data were excluded
Replication	The reproducibility was evaluated on the Delta / Omicron mixes that were extracted in duplicate to test the impact of PCR amplification prior to sequencing on co-infection characterization.<br>The reproductibility was also assessed on clinical samples.To confirm co-infections in patients and rule-out potential contamination during initial sequencing, all 64 samples with positive secondary lineage mutation ratio were re-extracted and sequenced in duplicate. In total, 53 samples had a positive secondary lineage mutation ratio in duplicate.
Randomization	Randomization was not relevant for this retrospective study with no predetermined groups.
Blinding	Blinding was not relevant for this retrospective study with no predetermined groups.

Reporting for specific materials, systems and methods

We require information from authors about some types of materials, experimental systems and methods used in many studies. Here, indicate whether each material, system or method listed is relevant to your study. If you are not sure if a list item applies to your research, read the appropriate section before selecting a response.

Materials & experimental systems

n/a	Involved in the study	
☒	☐	Antibodies
☒	☐	Eukaryotic cell lines
☒	☐	Palaeontology and archaeology
☒	☐	Animals and other organisms
☐	☒	Human research participants
☒	☐	Clinical data
☒	☐	Dual use research of concern

Methods

n/a	Involved in the study	
☒	☐	ChIP-seq
☒	☐	Flow cytometry
☒	☐	MRI-based neuroimaging

Human research participants

Policy information about studies involving human research participants

Population characteristics	The clinical samples selected for this study were samples collected from hospitalized patients, health care workers and outpatients. The covariate-relevant population characteristics of the human research participants are age and gender.
Recruitment	The clinical sample selection was based on random sequencing for Flash survey samples (outpatients and hospitalized patients) and systematic sequencing for hospitalized patients and health care workers of HCL. Only samples with a Ct-value < 28 were sequenced.
Ethics oversight	Samples used in this study were collected as part of an approved ongoing surveillance conducted by the NRC of HCL. The investigations were carried out in accordance with the General Data Protection Regulation (Regulation (EU) 2016/679 and Directive 95/46/EC) and the French data protection law (Law 78–17 on 06 January 1978 and Décret 2019–536 on 29 May 2019). Samples were collected for regular clinical management, with no additional samples for the purpose of this study.

Patients were informed of the research and their non-objection approval was confirmed. This study was approved by the ethics committee of the Hospices Civils de Lyon (HCL), Lyon, France and registered on the HCL database of RIPHN studies (AGORA N°41).

Note that full information on the approval of the study protocol must also be provided in the manuscript.
